# Molecular and evolutionary analysis of dengue virus serotype 2 isolates from Korean travelers in 2015

**DOI:** 10.1007/s00705-020-04653-z

**Published:** 2020-05-14

**Authors:** Eun-Ha Hwang, Green Kim, Hanseul Oh, You Jung An, Jiyeon Kim, Jung Heon Kim, Eung-Soo Hwang, Jong-Hwan Park, JungJoo Hong, Bon-Sang Koo

**Affiliations:** 1 National Primate Research Center, Korea Research Institute of Bioscience and Biotechnology, Cheongju, Republic of Korea; 2 Laboratory Animal Medicine, College of Veterinary Medicine, Chonnam National University, Gwangju, Republic of Korea; 3 Department of Microbiology and Immunology, Seoul National University College of Medicine, Seoul, Republic of Korea; 4 Institute of Endemic Diseases, Seoul National University Medical Research Center, Seoul, Republic of Korea

## Abstract

In Korea, dengue infection has been frequently reported in travelers to tropical and subtropical countries. Global warming increases the probability of autochthonous dengue outbreaks in Korea. In this report, the molecular and evolutionary properties of four dengue virus (DENV) type 2 isolates from Korean overseas travelers were examined. Three of these isolates were classified as Cosmopolitan genotypes and further divided into sublineages 1 (43,253, 43,254) and 2 (43,248), while the other isolate (KBPV-VR29) was related to American genotypes. The variable amino acid motifs related to virulence and replication were identified in the structural and non-structural proteins. A negative selection mechanism was clearly verified in all of the DENV proteins. Potential recombination events were identified in the NS5 protein of the XSBN10 strain. The substitution rate (5.32 × 10^−4^ substitutions per site) and the time of the most recent common ancestor (TMRCA) for each evolutionary group were determined by the Bayesian skyline coalescent method. This study shows that DENV type 2 strains with distinct phylogenetic, evolutionary, and virulence characteristics have been introduced into Korea by overseas travelers and have the potential to trigger autochthonous dengue outbreaks.

**Electronic supplementary material:**

The online version of this article (10.1007/s00705-020-04653-z) contains supplementary material, which is available to authorized users.

## Introduction

Dengue virus (DENV) is one of the most prevalent pathogens in tropical and subtropical countries [1]. Recently, the incidence and severity of this disease has dramatically increased worldwide. Before the 1970s, dengue outbreaks were reported in only nine countries. Recently, DENV epidemics have been observed in more than 100 countries [2]. A total of 3.6 billion people live in areas at risk for epidemic transmission, and nearly 400 million people suffer from DENV infection annually [1]. For this reason, dengue infection is one of the 17 diseases prioritized by the World Health Organization. This increased incidence has been caused by three factors: rapid urbanization, increased global travel, and global warming [3]. The virus can be transmitted to humans by mosquitoes of the species *Aedes aegypti* and *Aedes albopictus* [2]. Global warming has widened the growth habitats of these mosquito species and increased the distribution of dengue outbreaks worldwide [4].

DENV is a single-stranded, positive-sense arthropod RNA virus belonging to the genus *Flavivirus* of the family *Flaviviridae* [5]. The viral genome encodes three structural proteins (capsid [C], pre-membrane [prM], and envelope [E]) and seven non-structural proteins (non-structural [NS] 1, NS2A, NS2B, NS3, NS4A, NS4B, and NS5) [6]. DENV isolates are classified into four serotypes (DENV 1, 2, 3, and 4) and further divided into distinct genotypes based on 6–8% nucleotide and 3% amino acid sequence differences [6, 7]. DENVs within the same serotype generally share 65–70% nucleotide sequence identity [8]. DENV infections result in a wide spectrum of clinical signs, ranging from unapparent infection to severe dengue. Dengue fever is observed in most human cases and is characterized by a self-limiting fever. Dengue hemorrhagic fever (DHF) and dengue shock syndrome (DSS) can occur in severe dengue cases and are characterized by thrombocytopenia, increased vascular permeability, and hypovolemic shock [9].

Severe dengue seems to result from the complex interactions between the virus and the host immune system. Antibody-dependent enhancement (ADE) is a well-known mechanism leading to severe dengue infection. High titers of dengue antibody initially confer protection against heterogenous DENV serotypes, but after the titer decreases, the DENV antibody complexes exhibit enhanced virus binding to Fc gamma receptors of monocytes, leading to increased virus replication and virulence in the host [10]. In addition, several dengue virus strains have been more frequently associated with severe dengue cases [11–14]. For example, Asian DENV-2 genotypes tend to cause more-severe infections in humans than the American types. The clinical severity differs according to the interactions that occur between the virus and the host immune system. DENV-2NI-1 induces severe dengue in children with immunity to the DENV-1 serotype, but DENV-2NI-2B is virulent in children previously infected with the DENV-3 serotype [13].

DENV has been imported into Korea with increasing frequency by travelers coming from dengue-endemic countries [15, 16]. It has been reported *Ae. albopictus* is a secondary vector of DENV in Korea [17]. Recently, endemic outbreaks of dengue have been reported in Japan and Europe, which are located at a similar latitude to Korea [18–20]. Therefore, DENV carried by global travelers has the possibility of triggering dengue endemics in Korea. However, limited genome sequence information is available about DENV strains in Korea. In this report, we determined the full coding sequences of three DENV-2 viruses and performed molecular and evolutionary analysis.

## Materials and methods

### Viruses and RNA extraction

Three DENV-2 strains were provided from the National Culture Collection for Pathogens (Cheongju, Korea). These viruses were isolated from serum samples from Korean patients travelling to Singapore, India, and Thailand in 2015. The viruses were passaged twice in VERO-E6 cells, and the cell culture supernatant was stored at -80 °C until processing. Viral RNA was extracted from the supernatant using a QIAamp Viral RNA Mini Kit (QIAGEN, CA, USA) according to the manufacturer’s instructions and stored at -80 °C until use. One DENV-2 strain (KBPV-VR29) reported in Korea [21] was also evaluated in this study.

### RT-PCR and sequencing

Reverse transcription (RT)-PCR was performed for the amplification of the full coding region of each DENV isolate using a QIAGEN OneStep RT-PCR Kit (QIAGEN, CA, USA). The primers were described previously [22, 23], and their sequences are shown in Supplementary Table 1. The thermal cycling conditions were as follows: one cycle of reverse transcription (45℃ for 30 min), one cycle of initial denaturation (95℃ for 15 min), 40 cycles of amplification (94℃ for 10 s, 46℃ for 30 s, and 68℃ for 3 min), and final elongation (68℃ for 10 min). The PCR products were separated by 1% agarose gel electrophoresis and stained with SYBR Safe DNA Gel Stain Dye (Invitrogen, USA) and visualized by UV transillumination. Amplicons of the expected size were excised and purified using an Expin Gel SV Kit (GeneAll, Seoul, Korea). The nucleotide sequences were determined by direct sequencing using an ABI 3730XL sequencer (Macrogen, Seoul, Korea).

**Table 1:** Amino acid variations between the Korean isolates, the most similar strains, and the standard DENV 2SS

Strains	Reference strain		Structural protein				Non-structural protein							
		Substitution	C (114)	prM (166)	E (495)	E ^a^ (93)	NS1 (352)	NS2A (218)	NS2B (130)	NS3 (618)	NS4A (127)	Protein 2 K (23)	NS4B (248)	NS5 (900)
43,248	DENV2SS	Number	2	8	11	1	7	6	4	13	3	0	3	24
		Rate	98.25	95.18	97.78	98.92	98.01	97.25	96.92	97.9	97.64	100	98.79	97.34
	KY921905^b^	Number	0	0	0	0	9	0	0	1	0	0	1	7
		Rate	100	100	100	100	97.44	100	100	99.84	100	100	99.6	99.22
43,253	DENV2SS	Number	2	7	12	1	7	9	2	9	2	0	4	15
		Rate	98.25	95.78	97.78	98.92	98.01	95.87	98.46	98.54	98.43	100	98.39	98.33
	MH822954^b^	Number	0	0	1	0	1	0	0	0	0	0	1	0
		Rate	100	100	99.8	100	99.72	100	100	100	100	100	99.6	100
43,254	DENV2SS	Number	2	6	13	2	7	7	1	11	3	0	6	20
		Rate	98.25	96.39	97.37	97.85	98.01	96.79	99.23	98.22	97.64	100	97.58	97.78
	KY672952^b^	Number	0	0	1	0	7	2	0	0	0	0	2	0
		Rate	100	100	99.8	100	98.01	99.08	100	100	100	100	99.19	100
KBPV-VR29	DENV2SS	Number	6	4	10	1	9	9	2	18	3	0	7	22
		Rate	94.74	97.59	98.18	98.92	97.44	95.87	98.46	97.9	97.64	100	97.18	97.56
	JQ686088^b^	Number	0	0	1	0	0	1	0	2	0	0	2	2
		Rate	100	100	99.8	100	100	99.54	100	99.68	100	100	99.19	99.78

^a^Predicted domain 3 in the envelope protein (aa position 303–395)

^b^The most similar strain to each DENV-2 strain isolated in Korea

### Sequence and phylogenetic analysis

The nucleotide sequences of three DENVs were manipulated using BioEdit software, version 7.0.5.3, assembled in CLC Genomics Workbench 12.0 (QIAGEN, CA, USA), and aligned with DENV reference genes using the CLUSTAL W method. Phylogenetic analysis based on the complete coding region and the region encoding the envelope (E) protein of 122 DENVs, including the four Korean isolates from this study, was performed by the maximum-likelihood method, using the Tamura Nei model of gamma-distribution rates (TN93 + G + I model) with 1,000 bootstrap replicates in Molecular Evolutionary Genetics Analysis (MEGA, version 7.0.26) [24]. Reference viruses were randomly selected based on country, year of isolation, genotype, and sequence quality. Sequence similarities among DENV-2 isolates were analyzed using CLC Genomics Workbench 12.0. Amino acid substitutions and mutation rates of viruses were compared to those of a DENV 2SS strains (M29095) and the most similar DENV strains (KY921905, MH822954, KY672952, and JQ686088) identified by the NCBI BLAST programs.

### Bayesian evolutionary analysis

To estimate the nucleotide substitution rates and the time to the most recent common ancestor (TMRCA), a total of 100 nucleotide sequences from the E region of DENV-2 strains with known isolation dates and locations were retrieved from the GenBank database. Sequences with low quality, high similarity (99% identity), and recombinants were excluded from the evolutionary analysis. DENV datasets were analyzed using the Bayesian Markov Chain Monte Carlo (MCMC) method in the BEAST 2 package [25, 26]. The best-fit substitution models were selected based on the values of the Akaike Information Criterion and Bayesian Information Criterion using modeling test software in CLC Genomics Workbench. The dataset was evaluated using a relaxed uncorrelated lognormal molecular clock with a Bayesian skyline coalescent prior [27]. Procedures were run twice for 60,000,000 generations, and the parameter values were sampled after each 6,000 steps. The final log and tree files were combined using LogCombiner version 2.5.2, excluding 10% burn-in from each run. The combined results of the log files with an effective sample size (ESS) greater than 200 were analyzed and viewed using Tracer version 1.7.1 (https://tree.bio.ed.ac.uk/software/tracer/). The combined trees were annotated using Tree Annotator v.1.8.2 and visualized using the FigTree 1.4.2 program.

### Selection pressure analysis

Selection pressure was evaluated for 122 DENV sequences by four different methods, using the Datamonkey web server of the HyPhy package [28]. Sequences with ambiguous results and high similarity (> 99%) were excluded from this analysis. The ratio of synonymous to non-synonymous substitutions (ω ratio) was calculated using the parameters single-likelihood ancestor, fixed effects likelihood, mixed effects model of evolution, and fast, unconstrained Bayesian approximation [29–31]. Selection pressure analysis was performed for the structural proteins (C, prM, and E) and the non-structural proteins (NS1, NS2A, NS2B, NS3, NS4A, NS4B, and NS5). Sites under positive or negative selection were identified based on statistical significance (*p-*value < 0.1 or posterior probability < 0.9) using at least two methods.

### Recombination analysis

Recombination was investigated using seven different methods in the Recombination Detection Program (RDP) version 4.56 package (https://web.cbio.uct.ac.za/~darren/rdp.html) [32]. Putative recombination events were considered likely only if they were predicted with high statistical significance (*p*-value < 0.00001) by at least two methods. The following 12 DENV-2 strains were selected based on the phylogenetic analysis and isolation location for recombination analysis: Korean strains (43,248, 43,253, 43,254, and KBPV-VR29), KY474309 (Ecuador, American/Asian), GQ868542 (Thailand, Asian 1), HQ891023 (Taiwan, Asian 2), M29095 (Papua New Guinea, Asian 2), MF156242 (China, Cosmopolitan), MH822954 (India, Cosmopolitan), MK513444 (Singapore, Cosmopolitan), EU056811 (Peru, American), and FJ467493 (Malaysia, Sylvatic).

### Prediction of B-cell epitopes

B-cell epitopes within each ORF of isolates DENV 2SS, 43,248, 43,253, 43,254, and KBPV-VR29 were predicted using the BCPreds prediction tool [33]. The putative epitopes were further evaluated using the VaxiJen server (antigenicity prediction server). Regions with BCPreds scores of > 0.8 and VaxiJen scores of > 0.6 were identified as probable B-cell epitopes.

### Tertiary structure prediction

Prediction of proteins tertiary structure were done based on the amino acid sequences of the E protein of DENV-2 strain 43,248 using the protein homology/analogy recognition engine, version 2.0 [34]. The constructed structures were edited and visualized using the Jmol program, an open-source browser-based HTML5 viewer and stand-alone Java viewer for chemical structures in 3D (https://www.jmol.org/) [35].

## Results

### Sequencing and phylogenetic analysis

**Fig. 1:**
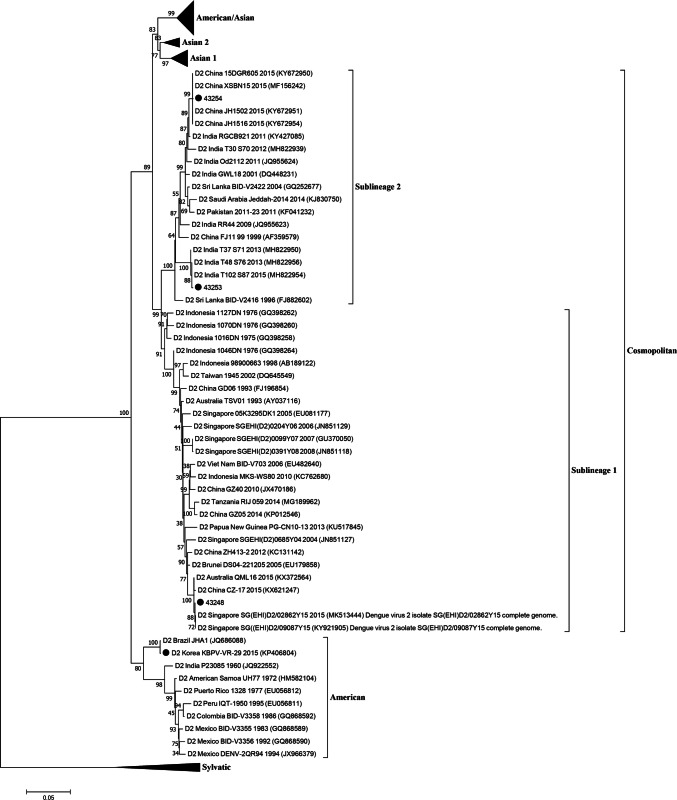
Phylogenetic analysis of DENV 2 viruses isolated in Korea based on full-coding regions. Black circles indicate dengue virus type 2 isolated in Korea. Four genotypes including American/Asian, Asian 1, Asian 2, and Sylvatic were compressed and expressed as the triangle shape

The whole genomes of three DENV isolates were successfully sequenced, and they were confirmed to be DENV-2 by nucleotide BLAST analysis. Phylogenetic analysis of 122 DENV-2 strains based on the full coding sequence (Fig. 1) and the E regions (Fig. S1) showed that three Korean isolates (43,248, 43,253, and 43,254) belonged to the Cosmopolitan genotype, and one strain in Korea (KBPV-VR29) belonged to the American genotype. Three viruses of the Cosmopolitan genotype were further divided into two sublineages: sublineage 1 (43,253 and 43,254) and sublineage 2 (43,248). The nucleotide sequence identity values of DENVs within the Cosmopolitan genotype were 95.2–99.9% within sublineage 1, 95.2–99.8% within sublineage 2, and 93.0–96.4% between the two sublineages. The most similar strains to the Korean isolates were Singapore 2015 (KY921905, 99.86%) to 43,248, India 2015 (MH822954, 99.9%) to 43,253, China 2015 (KY672952, 99.87%) to 43,254, and Brazil JHA-1 (JG686088, 99.78%) to KBPV-VR29. All sequence files are available in the GenBank database (accession numbers MK629884-MK629886).

### Sequence analysis

The amino acid sequence encoded by each ORF of the four Korean DENV isolates (43,248, 43,253, 43,254, and KBPV-VR29) were compared to the most similar strains of each virus and the DENV 2SS strain (Table 1). The most variable regions of DENV isolates of in the Cosmopolitan genotype compared to DENV 2SS were identified in the prM protein, but the KBPV-VR29 strain in the American genotype exhibited the lowest sequence similarity in the C protein. The E proteins commonly showed sequence identity values of over 97%. The results of nucleotide and amino acid sequence comparisons of E proteins between the Korean isolates and DENV 2SS are summarized in Table 2. In general, the amino acid sequence identity values were higher than the nucleotide sequence identity values.

**Table 2:** Percent nucleotide and deduced amino acid sequence identity of envelope genes of Korean isolates

	DENV 2SS	43,248	43,253	43,254	KBPV VR29
DENV 2SS		93.80	93.87	94.07	93.06
43,248	97.78		93.94	93.94	92.12
43,253	97.58	99.39		95.76	91.92
43,254	97.37	99.19	98.99		91.58
KBPV VR29	97.98	97.37	97.58	96.97	

The values below the diagonal indicate amino acid sequence identity (%), while the values above the diagonal indicate nucleotide sequence identity (%)

### Bayesian evolutionary analysis

**Fig. 2:**
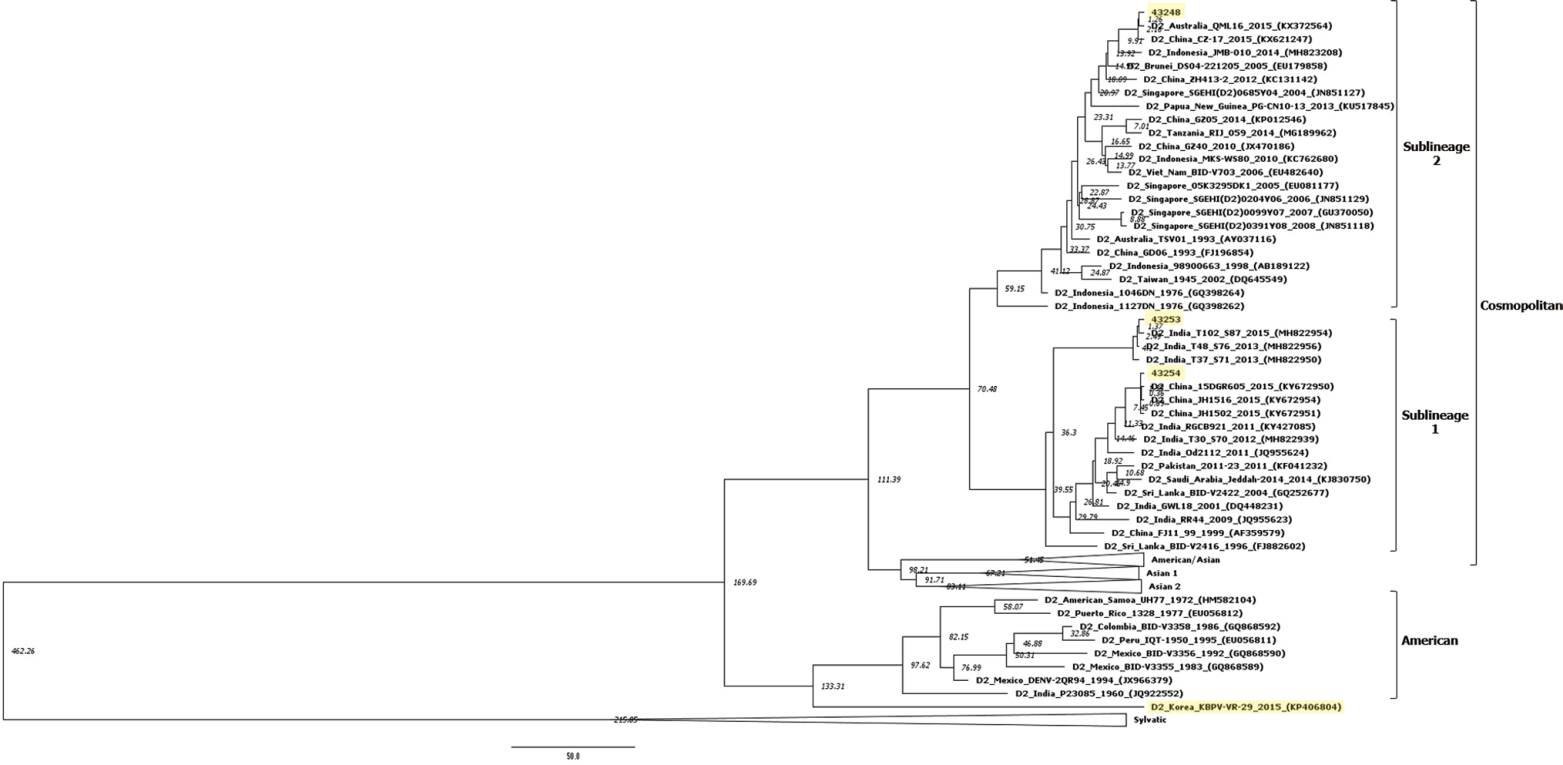
Bayesian evolutionary analysis of dengue viruses in Korea. The model was evaluated using a relaxed uncorrelated lognormal molecular clock with a prior Bayesian skyline coalescent. Yellow shaded strains indicate dengue virus type 2 isolated in Korea. Four genotypes including American/Asian, Asian 1, Asian 2, and Sylvatic were compressed and expressed as the triangle shape. The nodes indicate the node heights

For the 100 E genes of DENV-2 strains, the rate of nucleotide substitution and TMRCA with over 200 ESS values were obtained (Fig. 2). The rate of nucleotide substitutions was 5.32 × 10^–4^ (95% high probability density [HPD] interval, 4.11 × 10^–4^ to 6.69 × 10^–4^). The TMRCA of the Cosmopolitan genotype was found to be 70 years with a 95% HPD interval between 51 to 90 years (1945 [1925–1964]). The TMRCAs of sublineages 1 and 2 within the Cosmopolitan genotype were calculated to be 40 and 59 years, with 95% HPD intervals between 29 to 52 years (1975 [1963–1986]) and 46 to 75 years (1956 [1940–1969]), individually. The TMRCA of the American genotype was evaluated to be 133 years with a 95% HPD interval between 101 to 172 years (1882 [1843–1914]). The mean TMRCAs of the epidemic strains 43,248, 43,253, and 43,254 were 10, 4, and 7 years, respectively.

### Selection pressure

The results of selection pressure analysis for each viral protein of the 122 DENV-2 strains are presented in Table 5. Positive selection pressure was only identified in the three non-structural proteins NS2A, NS3, and NS5 proteins. The highest rates of negative selection pressure were identified in the NS3. Inversely, there was a significantly lower value of the C protein in DENVs compared to those of the other nine proteins.

### Recombination analysis

**Fig. 3:**
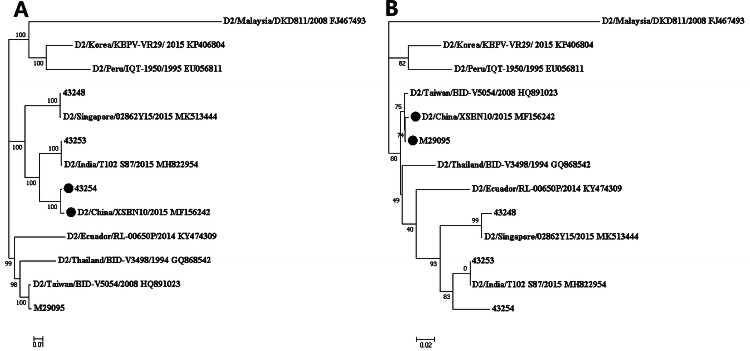
Recombinant events in the DENV2 strains. Phylogenetic analysis of DENV type 2 strains based on nucleotide positions (1 to 8891 and 9279 to 10,179) (**a**) and (8892 to 9278) (**b**) by the maximum likelihood method. Potential recombinant (MF156242), major parent (43254), and minor parent (M29095) were indicated as black circles

Two putative recombination events were identified using five methods (Table S2). The recombination breakpoints were within the NS5 gene. The nucleotide positions of the sequences from the minor parents (M29095 and unknown) were 8892 to 9278 and 8765 to 8840, respectively. A recombinant strain (MF156242) was composed of a major parent (43,254) and a minor parent (M29095) (Fig. 3).

## Discussion

Recently, there has been a continuous increase in DENV outbreaks worldwide due to global warming, increased global travel, and rapid urbanization [3, 4]. DENV infections have been increasingly identified in Korean travelers returning from dengue-endemic countries [15, 16]. However, information about DENV isolates from Korea has been limited. In this report, we present a molecular and evolutionary analysis of DENV type 2 strains isolated from Korean overseas travelers.

The DENV-2 strains were classified into six distinct genotypes including Asian 1, Asian 2, Cosmopolitan, American, Asian/American, and sylvatic [6, 40]. In phylogenetic analysis, three viruses (43,248, 43,253, and 43,254) were classified as the Cosmopolitan genotype, and one strain (KBPV-VR29) was classified as the American genotype. Three viruses of the Cosmopolitan genotype were further divided into two sublineages, namely sublineage 1 and sublineage 2 (Fig. 1). The nucleotide sequences within the same sublineage were more than 95.2% identical, but those of members of different sublineages were only 93.0%–96.4% identical. In previous reports, genotypes of DENV were distinguished based on 6–8% nucleotide sequence divergence [6, 7]. Therefore, a new genotype classification should be considered for the Cosmopolitan genotype.

The four DENV-2 strains exhibited similarities to epidemic strains from other countries. The discovery that the 43,253 strain clustered with endemic DENV-2 strains was taken as a warning sign for in New Delhi, India, which has faced continued dengue outbreaks every 3–4 years [37, 41]. The 43,254 strain, isolated from a Korean travelling to China, clustered with DENV type 2 epidemic strains isolated in Yunnan province in China [42, 43]. The strains were prevalent in this area and caused more than 1,000 cases of dengue during the second half of 2015. These viruses seemed to have evolved from DENV strains from India in 2011 and 2012 (Fig. 1). The 43,248 strain proved to be similar to strains endemic in Thailand [44]. Only the KBPV-VR29 strain belonged to the American genotype and exhibited the most similarity to the JHA-1 strain, which is highly neurovirulent in mice [38].

**Fig. 4:**
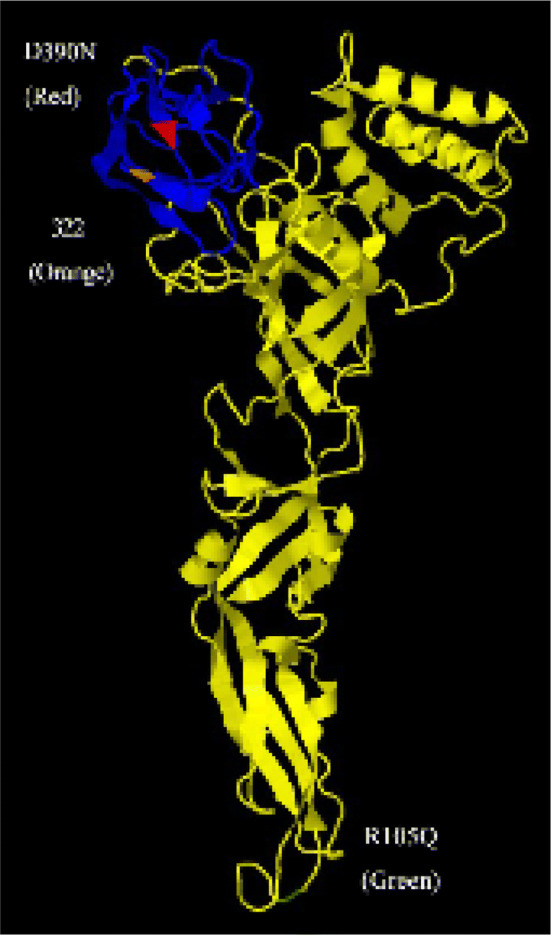
The structural motifs in the predicted tertiary envelope proteins of the dengue virus. The colored regions indicate amino acid substitution related to increased virulence (D390N in red, R105Q in green) and the motif related to attachment and fusion (322 in orange). Blue colored regions indicate the predictive domain 3 portion of envelope proteins

Severe dengue is a life-threatening clinical form of dengue infection. The factors affecting dengue severity include the ADE phenomenon, the virulence of the genotype, and replication capacity of the virus [10, 11, 41, 45]. Therefore, B-cell epitopes and the motifs related to viral replication and pathogenicity were compared among the strains. High titers of DENV have been shown to be related to severe dengue infection in humans [41, 45]. The DENV isolates described in this report possessed variable motifs and showed evidence of recombination in the NS5 region, which encodes the RNA-dependent RNA polymerase and therefore could affect replication capacity (Table 3, Fig. 3) [36–39]. Three motifs in the E protein were differentiated among the Korean isolates (Table 3). Two of them were located in domain III of the E protein, which is important for determining host range, tissue tropism, and virulence (Fig. 4) [46]. Therefore, the DENV isolates from this study are expected to differ in their virulence and replication capacity, especially the KBPV-VR29 strain.

**Table 3:** Amino acids predicted to be involved in DENV replication and virulence

Protein	Amino acid position	Strains			
		43,248	43,253	43,254	KBPV-VR29
PrM	28	E	E	E	K
E	105	R	R	R	Q
	291	K	K	K	K
	295	K	K	K	K
	322	I	I	V	I
	390	S	S	S	N
	402	F	F	F	F
	404	T	T	T	T
NS1	105	R	R	R	Q
	128	H	H	H	L
NS3	567	V	V	I	T
NS4B	17	S	S	S	S
NS5	271	V	T	T	I
NS5	645	N	D	D	N
NS5	676	N	G	S	R
NS5	800	T	K	R	S
NS5	819	Q	Q	Q	Q

The ADE phenomenon is known to play a role in severe dengue [47]. Antibodies with low affinity and titer can promote the entry of DENV into immune cells expressing the *Fc* gamma receptor, such as myeloid and mast cells, causing increased virus replication and severe dengue infection [10, 47]. Amino acid sequence differences within B-cell epitopes of DENVs might be more directly involved in ADE than other genomic regions. In this study, we found amino acid differences in all predicted B-cell epitopes between the viral proteins of members of the Cosmopolitan and American genotypes (Table 4). Therefore, severe dengue could be manifested in Koreans by serial infection with imported DENVs.

**Table 4:** Amino acid differences in predicted B-cell epitopes among dengue viruses isolated in Korea and the standard DENV 2SS

Protein	Position	Strains					Predictive B cell epitope (sequence position)*
		DENV 2SS	43,248	43,253	43,254	KBPV- VR29	
prM	80	T	A	A	A	T	CWCNSTSTWVTYGTCTTTGE (66–85)
E	71	D	A	A	A	D	KLTNTTTDSRCPTQGEPSLN (63–82)
	182	T	T	T	V	T	LTGYGTVTMECSPRTGLDFN (174–193)
	363	S	S	S	S	R	TVNPIVTEKDSPVNIEAEPP (352–371)
	390	N	S	S	S	N	VEPGQLKLSWFKKGSSIGQM (381–400)
NS1	99	A	A	A	A	V	VKLTIMTGDIKGIMQAGKRS (84–103)
	105	Q	R	R	R	Q	QPQPTELKYSWKTWGKAKML (105–124)
	112	K	K	K	K	R	AGPVSQHNYRPGYHTQTAGP (248–267)

*The numbering of amino acids is based on DENV 2SS

The nucleotide substitution rate in the E protein was found in this study to be 5.32 × 10^–4^, which is within the 95% HPD intervals of the rates given in a previous report [37]. The substitution rates per sites range from 10^–8^ to 10^–6^ in DNA viruses and from 10^–6^ to 10^–4^ in RNA viruses [48]. DENV has an especially high substitution rate among RNA viruses. The estimated TMRCAs of the isolates 43,248, 43,253, and 43,254 were 10, 4, and 7 years, respectively. DENV has evolved within 10 years and has caused outbreaks in various regions of the world. Evidence of negative selection pressure was found in all of the proteins of DENV (Table 5). Positive pressure was not found in the E protein, which contains important epitopes for neutralizing antibodies [6]. Viruses can escape host immunity by altering their viral proteins for survival in the host. These mutations are primarily caused by immune pressure in natural infection [49, 50]. Thus, sites under positive selection are mainly found in and near epitopes of HA proteins of influenza viruses [49]. Interestingly, positive selection was found in non-structural proteins of DENV, including NS2A, NS3, and NS5 (Table 5). These proteins appear to have been subjected to immune pressure in the host.

**Table 5:** Selection pressure analysis of the dengue viruses isolated in Korea

Type of selection pressure	Structural protein			Non-structural protein						
	C (114)	prM (166)	E (495)	NS1 (352)	NS2A (218)	NS2B (128)	NS3 (618)	NS4A (127)	NS4B (246)	NS5 (900)
Positive										
Number	0	0	0	0	1	0	1	0	0	3
Rates (%)	0	0	0	0	0.5	0	0.2	0	0	0.3
Negative										
Number	51	121	360	251	160	98	487	100	192	649
Rates (%)	44.7	72.9	72.7	71.3	73.4	76.6	78.8	78.7	78.0	72.1

*Ae. albopictus*, which can serve as a second vector for dengue infection, represents a large proportion of the mosquito species in Korea [17]. Recently, global dengue outbreaks were reported in China (27.9%), Singapore (27.0%), and Malaysia (15.1%). The majority of dengue cases have been reported in the Western Pacific region (72.4%) [4], which has been a popular destination for Korean travelers. The numbers of Korean travelers were 3,854,869 to China, 1,717,867 to Thailand, and 111,076 to India according to the Korean Tourism Organization. A previous report stated that global warming has increased the probability of domestic DENV outbreaks in Korea [51]. Recently, autochthonous dengue infections have been reported in temperate countries, including Japan and France [18–20]. The overall epidemiological situation has increased the likelihood of dengue outbreaks in Korea.

In this study, we found that DENV strains with different having molecular, phylogenetic, evolutionary, and virulence characteristics have been introduced into Korea by travelers. Considering that dengue infections usually cause inapparent or mild symptoms, the actual incidence of dengue infection in Korean travelers could be significantly higher than previously reported [15, 16]. Therefore, active surveillance of DENV infection should be performed for screening Korean travelers returning from tropical and subtropical countries.

## Electronic supplementary material

Below is the link to the electronic supplementary material.

Supplementary file1 Phylogenetic analysis of DENV-2 isolates from Korea based on the envelope genes. Black circles indicate dengue virus type 2 isolated in Korea. Four genotypes including American/Asian, Asian 1, Asian 2, and Sylvatic were compressed and expressed as a triangle (TIF 38271 kb)

Supplementary file2 Primers targeting different genetic regions of dengue virus type 2 (XLSX 11 kb)

Supplementary file3 Recombination analysis of DENV 2 isolates in Korea (XLSX 10 kb)

## References

[1] Bhatt S, Gething PW, Brady OJ, Messina JP, Farlow AW, Moyes CL, Drake JM, Brownstein JS, Hoen AG, Sankoh O (2013). The global distribution and burden of dengue. Nature.

[2] WH Organization (2014). Dengue and severe dengue.

[3] Gubler D (2012). Dengue, urbanization and globalization: the unholy trinity of the 21st century. Int J Infect Dis.

[4] Guo C, Zhou Z, Wen Z, Liu Y, Zeng C, Xiao D, Ou M, Han Y, Huang S, Liu D (2017). Global epidemiology of dengue outbreaks in 1990–2015: a systematic review and meta-analysis. Front Cell Infect Microbiol.

[5] Lindenbach BD, Rice CM (2003). Molecular biology of flaviviruses. Adv Virus Res.

[6] Weaver SC, Vasilakis N (2009). Molecular evolution of dengue viruses: contributions of phylogenetics to understanding the history and epidemiology of the preeminent arboviral disease. Infect Genet Evol.

[7] Rico-Hesse R (1990). Molecular evolution and distribution of dengue viruses type 1 and 2 in nature. Virology.

[8] Green S, Rothman A (2006). Immunopathological mechanisms in dengue and dengue hemorrhagic fever. Curr Opin Infect Dis.

[9] Monath TP (1994). Dengue: the risk to developed and developing countries. Proc Natl Acad Sci.

[10] Halstead SB (2007). Dengue. Lancet.

[11] Williams M, Mayer SV, Johnson WL, Chen R, Volkova E, Vilcarromero S, Widen SG, Wood TG, Suarez-Ognio L, Long KC (2014). Lineage II of Southeast Asian/American DENV-2 is associated with a severe dengue outbreak in the Peruvian Amazon. Am J Trop Med Hyg.

[12] Rico-Hesse R, Harrison LM, Salas RA, Tovar D, Nisalak A, Ramos C, Boshell J, de Mesa MTR, Nogueira RM, da Rosa AT (1997). Origins of dengue type 2 viruses associated with increased pathogenicity in the Americas. Virology.

[13] OhAinle M, Balmaseda A, Macalalad AR, Tellez Y, Zody MC, Saborío S, Nuñez A, Lennon NJ, Birren BW, Gordon A (2011). Dynamics of dengue disease severity determined by the interplay between viral genetics and serotype-specific immunity. Sci Transl Med.

[14] Soo K-M, Khalid B, Ching S-M, Chee H-Y (2016). Meta-analysis of dengue severity during infection by different dengue virus serotypes in primary and secondary infections. PLoS ONE.

[15] Yeom J-S (2017). Current status and outlook of mosquito-borne diseases in Korea. J Korean Med Assoc.

[16] Park J-H, Lee D-W (2012). Dengue fever in South Korea, 2006–2010. Emerg Infect Dis.

[17] Yang S, Lee E, Lee W, Shin-Hyeong C (2018). Geographical distribution of *Aedes albopictus* around urban areas in Korea. Public Health Weekly Rep.

[18] Tsuda Y, Maekawa Y, Ogawa K, Itokawa K, Komagata O, Sasaki T, Isawa H, Tomita T, Sawabe K (2016). Biting density and distribution of *Aedes albopictus* during the September 2014 outbreak of dengue fever in Yoyogi Park and the vicinity in Tokyo Metropolis, Japan. Jpn J Infect Dis.

[19] Kutsuna S, Kato Y, Moi ML, Kotaki A, Ota M, Shinohara K, Kobayashi T, Yamamoto K, Fujiya Y, Mawatari M (2015). Autochthonous dengue fever, Tokyo, Japan, 2014. Emerg Infect Dis.

[20] Succo T, Leparc-Goffart I, Ferré J-B, Roiz D, Broche B, Maquart M, Noel H, Catelinois O, Entezam F, Caire D (2016). Autochthonous dengue outbreak in Nîmes, south of France, July to September 2015. Eurosurveillance.

[21] Go YY, Jung E, Balasuriya UB (2015). Complete genome sequences of three laboratory strains of dengue virus (serotypes 2, 3, and 4) available in South Korea. Genome Announc.

[22] Christenbury JG, Aw PP, Ong SH, Schreiber MJ, Chow A, Gubler DJ, Vasudevan SG, Ooi EE, Hibberd ML (2010). A method for full genome sequencing of all four serotypes of the dengue virus. J Virol Methods.

[23] Cruz CD, Torre A, Troncos G, Lambrechts L, Leguia M (2016). Targeted full-genome amplification and sequencing of dengue virus types 1–4 from South America. J Virol Methods.

[24] Kumar S, Stecher G, Tamura K (2016). MEGA7: molecular evolutionary genetics analysis version 7.0 for bigger datasets. Mol Biol Evol.

[25] Bouckaert R, Heled J, Kühnert D, Vaughan T, Wu C-H, Xie D, Suchard MA, Rambaut A, Drummond AJ (2014). BEAST 2: a software platform for Bayesian evolutionary analysis. PLoS Comput Biol.

[26] Drummond AJ, Suchard MA, Xie D, Rambaut A (2012). Bayesian phylogenetics with BEAUti and the BEAST 1.7. Mol Biol Evol.

[27] Drummond AJ, Rambaut A, Shapiro B, Pybus OG (2005). Bayesian coalescent inference of past population dynamics from molecular sequences. Mol Biol Evol.

[28] Weaver S, Shank SD, Spielman SJ, Li M, Muse SV, Kosakovsky Pond SL (2018). Datamonkey 2.0: a modern web application for characterizing selective and other evolutionary processes. Mol Biol Evol.

[29] Kosakovsky Pond SL, Frost SD (2005). Not so different after all: a comparison of methods for detecting amino acid sites under selection. Mol Biol Evol.

[30] Murrell B, Wertheim JO, Moola S, Weighill T, Scheffler K, Pond SLK (2012). Detecting individual sites subject to episodic diversifying selection. PLoS Genet.

[31] Murrell B, Moola S, Mabona A, Weighill T, Sheward D, Kosakovsky Pond SL, Scheffler K (2013). FUBAR: a fast, unconstrained bayesian approximation for inferring selection. Mol Biol Evol.

[32] Martin DP, Murrell B, Golden M, Khoosal A, Muhire B (2015). RDP4: Detection and analysis of recombination patterns in virus genomes. Virus Evol.

[33] El-Manzalawy Y, Dobbs D, Honavar V (2008). Predicting linear B-cell epitopes using string kernels. J Mol Recognit.

[34] Kelley LA, Sternberg MJ (2009). Protein structure prediction on the Web: a case study using the Phyre server. Nat Protoc.

[35] Jmol J (2013) An open-source Java viewer for chemical structures in 3D. Jmol web page. https://www.jmol.org/. Accessed 15

[36] Waman VP, Kolekar P, Ramtirthkar MR, Kale MM, Kulkarni-Kale U (2016). Analysis of genotype diversity and evolution of Dengue virus serotype 2 using complete genomes. PeerJ.

[37] Afreen N, Naqvi IH, Broor S, Ahmed A, Kazim SN, Dohare R, Kumar M, Parveen S (2016). Evolutionary analysis of dengue serotype 2 viruses using phylogenetic and Bayesian methods from New Delhi, India. PLoS Negl Trop Dis.

[38] Kar M, Nisheetha A, Kumar A, Jagtap S, Shinde J, Singla M, Saranya M, Pandit A, Chandele A, Kabra SK (2018). Isolation and molecular characterization of Dengue virus clinical isolates from pediatric patients in New Delhi. Int J Infect Dis.

[39] Zhao Y, Li L, Ma D, Luo J, Ma Z, Wang X, Pan Y, Chen J, Xi J, Yang J (2016). Molecular characterization and viral origin of the 2015 dengue outbreak in Xishuangbanna, Yunnan, China. Sci Rep.

[40] Jiang L, Ma D, Ye C, Li L, Li X, Yang J, Zhao Y, Xi J, Wang X, Chen J (2018). Molecular characterization of dengue virus serotype 2 cosmospolitan genotype from 2015 dengue outbreak in Yunnan, China. Front Cell Infect Microbiol.

[41] Phadungsombat J, Lin MY-C, Srimark N, Yamanaka A, Nakayama EE, Moolasart V, Suttha P, Shioda T, Uttayamakul S (2018). Emergence of genotype Cosmopolitan of dengue virus type 2 and genotype III of dengue virus type 3 in Thailand. PLoS ONE.

[42] Amorim JH, Bizerra RSP, dos Santos Alves RP, Sbrogio-Almeida ME, Levi JE, Capurro ML, de Souza Ferreira LC (2012). A genetic and pathologic study of a DENV2 clinical isolate capable of inducing encephalitis and hematological disturbances in immunocompetent mice. PLoS ONE.

[43] Ben-Shachar R, Koelle K (2018). Transmission-clearance trade-offs indicate that dengue virulence evolution depends on epidemiological context. Nat Commun.

[44] Leitmeyer KC, Vaughn DW, Watts DM, Salas R, Villalobos I, Ramos C, Rico-Hesse R (1999). Dengue virus structural differences that correlate with pathogenesis. J Virol.

[45] Watterson D, Kobe B, Young PR (2012). Residues in domain III of the dengue virus envelope glycoprotein involved in cell-surface glycosaminoglycan binding. J Gen Virol.

[46] Rey FA, Heinz FX, Mandl C, Kunz C, Harrison SC (1995). The envelope glycoprotein from tick-borne encephalitis virus at 2 Å resolution. Nature.

[47] Katzelnick LC, Gresh L, Halloran ME, Mercado JC, Kuan G, Gordon A, Balmaseda A, Harris E (2017). Antibody-dependent enhancement of severe dengue disease in humans. Science.

[48] Sanjuán R, Nebot MR, Chirico N, Mansky LM, Belshaw R (2010). Viral mutation rates. J Virol.

[49] Duvvuri VR, Duvvuri B, Cuff WR, Wu GE, Wu J (2009). Role of positive selection pressure on the evolution of H5N1 hemagglutinin. Genom Proteom Bioinform.

[50] Chong Y, Ikematsu H (2018). Is seasonal vaccination a contributing factor to the selection of influenza epidemic variants?. Hum Vaccin Immunother.

[51] Lee H, Kim JE, Lee S, Lee CH (2018). Potential effects of climate change on dengue transmission dynamics in Korea. PLoS ONE.

