# Supplementary figures and images for: Molecular and evolutionary analysis of dengue virus serotype 2 isolates from Korean travelers in 2015
Source: Arch Virol. 2020 May 14;165(8):1739–48. doi: 10.1007/s00705-020-04653-z (PMC7351809; doi:10.1007/s00705-020-04653-z)

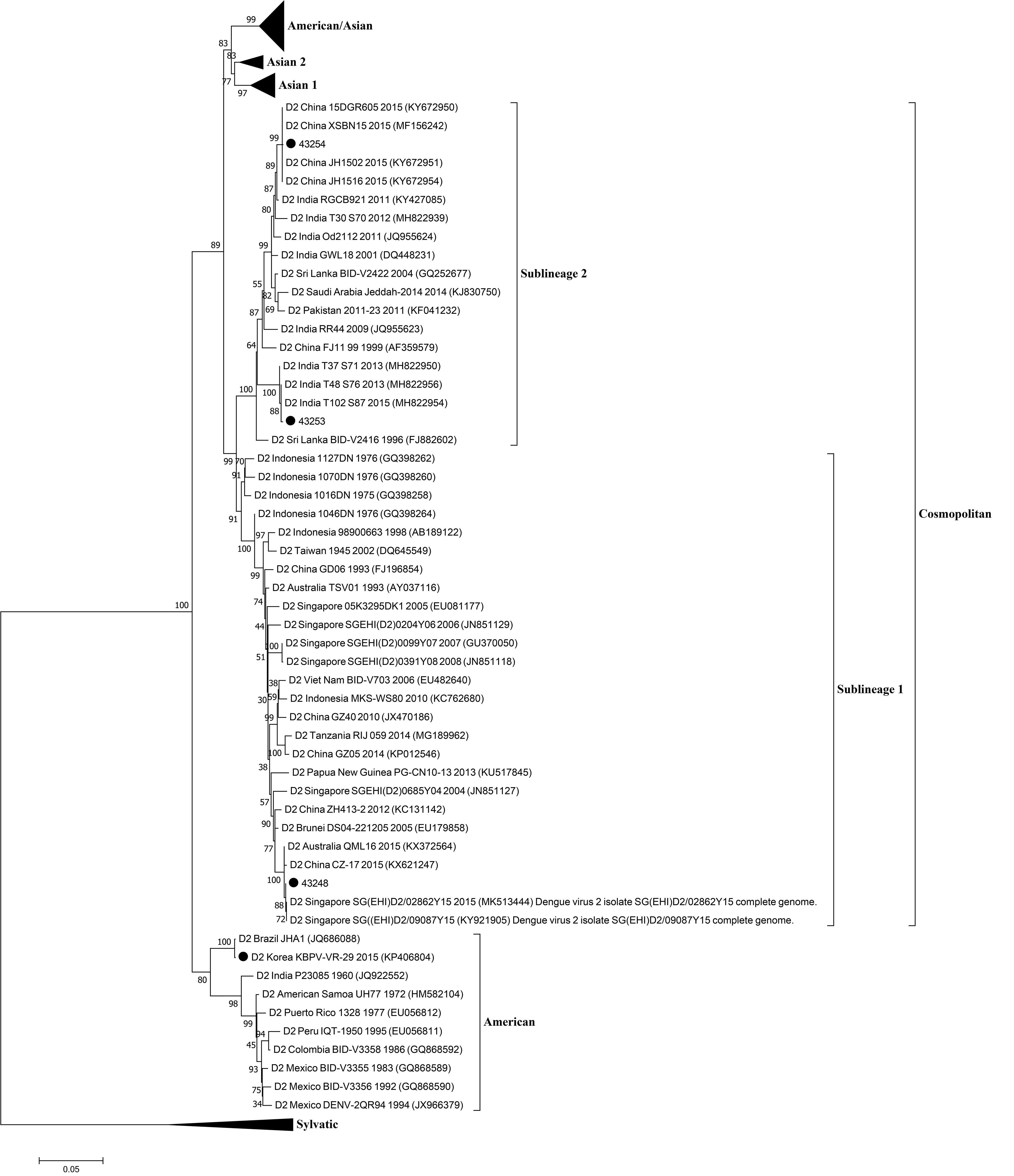

Supplement: Supplementary file 1 — Supplementary file1 Phylogenetic analysis of DENV-2 isolates from Korea based on the envelope genes. Black circles indicate dengue virus type 2 isolated in Korea. Four genotypes including American/Asian, Asian 1, Asian 2, and Sylvatic were compressed and expressed as a triangle (TIF 38271 kb) [file 705_2020_4653_MOESM1_ESM.tif]
